# American tegumentary leishmaniasis caused by *Leishmania* (*Viannia*) *braziliensis:* assessment of parasite genetic variability at intra- and inter-patient levels

**DOI:** 10.1186/1756-3305-6-189

**Published:** 2013-06-20

**Authors:** Fernanda S Oliveira, Cláudia M Valete-Rosalino, Sandro JB Pacheco, Filipe A Carvalho Costa, Armando O Schubach, Raquel S Pacheco

**Affiliations:** 1 Laboratório de Epidemiologia e Sistemática Molecular, Instituto Oswaldo Cruz, Fundação Oswaldo Cruz (IOC/FIOCRUZ), Av. Brasil, 4365, Rio de Janeiro, Rio de Janeiro, CEP 21040-900, Brazil; 2 Laboratório de Vigilância em Leishmanioses, Instituto de Pesquisa Clínica Evandro Chagas, Fundação Oswaldo Cruz (IPEC/FIOCRUZ), Rio de Janeiro, Brazil; 3 Departamento de Otorrinolaringologia e Oftalmologia, Faculdade de Medicina, Universidade Federal do Rio de Janeiro, Rio de Janeiro, Brazil

**Keywords:** *Leishmania* (*Viannia*) *braziliensis*, ATL, Genetic variability, LSSP-PCR, Numerical taxonomy

## Abstract

**Background:**

The genetic variability of *Leishmania* (*Viannia*) *braziliensis* was assessed at intra and interpatient levels of individuals with different clinical manifestations of American tegumentary leishmaniasis (ATL).

**Methods:**

Fifty-two samples, of which 13 originated from cutaneous lesions and 39 from mucosal lesions, provided by 35 patients, were examined by low-stringency single-specific-primer PCR (LSSP-PCR) and phenetic analysis. Genetic variability of *L*. (*V*.) *braziliensis*, in kinetoplast DNA (kDNA) signatures, was compared both from different patients and from different lesions of the same patient. Phenetic analysis was performed to evaluate the degree of heterogeneity of the kDNA minicircles. In order to evaluate inter and intrapatient *L*. (*V*.) *braziliensis* genetic variability, the percentage of shared bands and analysis of the coefficients of similarity were analyzed.

**Results:**

Different genetic profiles, representing kDNA signatures of the parasite, were obtained by LSSP-PCR analysis of each sample. Phenetic analysis grouped genetic profiles of different levels of differentiation from more similar to most divergent. The percentage of shared bands at the inter and intrapatient levels was 77% and 89%, respectively. Comparison of the average inter and intrapatient coefficients of similarity and their standard deviations were statistically significant (p < 0.001).

**Conclusion:**

Genetic variability at the intrapatient level was less pronounced than that between different patients. A conceptual model was proposed to better understand the complexity at both levels.

## Background

Leishmaniasis is a disease ranging from potential self-healing cutaneous lesions to mucosal disorders to more severe visceral affliction possibly resulting in death, all depending upon the species of *Leishmania*, the protozooan etiological agent.

American tegumentary leishmaniasis (ATL) caused by *Leishmania* (*Viannia*) *braziliensis* is characterized by chronicity, latency and metastatic tendencies. In some cases, after the appearance of an initial cutaneous lesion, multiple cutaneous lesions (disseminated leishmaniasis, DL) and/or mucosal lesions (mucosal leishmaniasis, ML) can arise as a consequence of the dissemination of the parasite through the blood and lymphatic systems [1-3]. Cutaneous lesions can remain active for many years and can coexist with mucosal lesions, as exhibited in the mucocutaneous leishmaniasis (MCL) form [4]. Less than 5% of cutaneous leishmaniasis cases develop destructive lesions in the upper respiratory tract, primarily in the nasal mucosa [5], and more than 25% of the DL cases present damaged mucosa simultaneously with cutaneous manifestations [6]. However, approximately 16% of the ML cases do not present any previous history of an initial cutaneous lesion [7].

Factors such as the immunological response of the patient and the genetic constitution of the parasite could influence the clinical manifestations and prognosis of this disease. It is also quite possible that these factors play a role in parasite persistence. Parasite persistence and reactivation have been reported to be contributing factors towards recurring episodes of ATL [8-10]. However, the mechanism of the dissemination and emergence of *L*. (*V*.) *braziliensis* to new cutaneous and mucosal tissue, the pathogenic process as well as the natural history of the recurrent leishmaniasis is not yet fully understood.

Human infection by *L*. (*V*.) *braziliensis* produces a broad spectrum of clinical manifestations that can be attributed to intraspecific variability of the parasite. The contribution of the parasite to the diversity of ATL patient clinical conditions has been examined in several studies aiming to investigate whether certain species and/or clonal populations of *Leishmania* are correlated with any clinical manifestations of the disease. Intraspecific heterogeneity among members of the subgenera *Viannia* and *Leishmania* was detected by genetic and molecular studies, which have afforded a better understanding of their epidemiology in diverse endemic areas [11-14].

Different polymorphic molecular markers based on the polymerase chain reaction (PCR) have been used in several studies of the *L*. (*V*.) *braziliensis* genetic variability and molecular epidemiology. The importance of this particular species was demonstrated by the existence of various intraspecific genetic polymorphisms and the variability of the clinical manifestations observed in ATL. Previous studies have established a correlation between genetic polymorphisms and the eco-epidemiological data of isolates [15-17]. Such studies reinforce the clonal population structure of *Leishmania*, as proposed by Tibayrenc *et al*. [18]. *Leishmania* species naturally circulate as a group of heterogeneous subpopulations, and demonstration of the polyclonality of the initial inoculum of multiple strains has been reported [19]. These mechanisms could influence the clinical prognosis of the disease and the efficiency of therapeutic strategies.

Our group recently introduced the application of low-stringency single-specific-primer PCR (LSSP-PCR) towards the investigation of intraspecific polymorphisms in the variable region of the kinetoplast DNA (kDNA) minicircles of *L*. (*V*.) *braziliensis* in ATL patients [20,21]. The purpose of the present study is to further investigate parasite subpopulations, with a particular focus on the genetic variability of *L*. (*V*.) *braziliensis* at intra and interpatient levels, using the same molecular marker associated with phenetic analyses. Towards this end, samples obtained from cutaneous and mucosal lesions of patients with different clinical manifestations of ATL from Rio de Janeiro, Brazil were analyzed.

## Methods

### Patients

This study included 35 patients from Rio de Janeiro, Brazil, presenting ATL and mucosal involvement, each attended at the Laboratório de Vigilância em Leishmanioses/Lab. VigiLeish, Instituto de Pesquisa Clínica Evandro Chagas – IPEC/Fiocruz, Rio de Janeiro, from January 2005 to December 2009. Patients were grouped according to their clinical manifestations in the following three categories: i) mucocutaneous leishmaniasis (MCL) for patients that presented cutaneous and mucosal lesions in contiguous and concomitant forms, ii) mucosal leishmaniasis (ML) for patients that presented only mucosal lesions (nasal and/or oral) and iii) disseminated leishmaniasis (DL) for patients presenting more than 10 cutaneous lesions and mucosal involvement. All patients were subjected to dermatological and otolaryngological examination, including direct inspection of the upper respiratory tract using rigid optic Karl Storz 70° and 30°, to assess, respectively, the presence or absence of larynx and pharyngeal or nasal lesions. This study was approved by the Ethical Committee of Research at IPEC/FIOCRUZ (process 0016.0.009-02), and all of the patients signed consent forms.

### Molecular diagnosis by PCR and low-stringency single-specific-primer PCR (LSSP-PCR) reactions

Patient biopsy samples from cutaneous and/or mucosal lesions were processed for molecular analysis. Extraction of DNA and molecular diagnostic PCR-based assays were performed as previously described [21,22]. The 750 bp fragments generated by specific PCR were purified with a Wizard PCR Prep System (Promega, Madison, WI) according to the manufacturer’s instructions. The purified products were then submitted to a second round of amplification (LSSP-PCR) with only one specific primer under low stringency conditions. Reaction conditions and visualization of the products were performed as described by Oliveira *et al*. [21].

### Phenetic analysis

Bands varying in size from 350–750 bp were selected for phenetic analysis. Analysis of the profiles generated by LSSP-PCR was performed by calculating the levels of similarity using the Simple Matching Coefficient (Sm). The matrix of similarity was transformed into a dendrogram through the Unweighted Pair Group Method Arithmetical Average (UPGMA) algorithm with the NTSYS version 2.1 program (Exeter Software).

### Comparison of inter and intrapatient genetic variability

Interpatient genetic variability was evaluated by: i) obtaining the coefficients of similarity between the patient sample and all of the other samples (from all of the patients), ii) calculating the arithmetic averages and their respective standard deviations of those interpatient coefficients of similarity and iii) calculating the average of those mean values, with the aim of estimating the average interpatient similarity (i.e. the variability existing among parasites recovered from lesions of different patients). Intrapatient genetic variability was estimated by calculating the average of the mean coefficient of similarity of each patient, taking into account the cutaneous and mucosal lesions present in the same patient. The averages and standard deviations (SD) of the inter and intrapatient coefficients of similarity were compared applying the stu*d*ent’s t-test.

Comparison of inter- and intra-patient similarities was also performed by analyzing the percentages of shared characters (bands), defined by genetic profiles generated by LSSP-PCR. The sharing frequency of each character between interpatient samples was calculated after first calculating the average of each sharing frequency. The same analysis was performed to compare different lesions from the same patient.

## Results

### Clinical-epidemiological characteristics of the patients

The clinical forms of ATL exhibited by the patients were: 16 with MCL, 17 with ML and 2 with DL. Of these patients, 25 (71.4%) were male and 10 (28.6%) were female. Patient age varied from 12 to 77 years. The presence of lesions in the nasal mucosa was observed in 77% of the patients. The two patients with DL (patients 34 and 35) exhibited concomitant cutaneous and mucosal lesions. Only one patient with MCL (patient 5) displayed concomitant nasal and oral mucosal lesions. Of the 17 patients with ML, 12 reported the absence of an initial cutaneous lesion or scar suggestive of leishmaniasis. Data are shown in Table 1.

**Table 1:** Clinical and epidemiological characteristics of the 35 patients analysed in this study

**Patient code**	**Age (years)**	**Sex**	**Clinical form**	**Location of lesion/Code**	
				**Cutaneous**	**Mucosal**
1	52	M	MCL	Arm/1MCL-C	Oral / 1MCL-O
2	48	M	MCL	Nostril/2MCL-C	Oral/2MCL-O
3	25	M	MCL	Arm/3MCL-C	Nasal/3MCL-N
4	54	M	MCL	ND	Oral/4MCL-O
5	47	M	MCL	Arm/5MCL-C	Nasal/5MCL-N
					Oral/5MCL-O
6	16	M	MCL	ND	Nasal/6MCL-N
7	50	M	MCL	Face/7MCL-C	Nasal/7MCL-N
8	29	M	MCL	Trunk/8LMC-C	Nasal/8MCL-N
9	55	M	MCL	ND	Oral/9MCL-O
10	42	M	MCL	ND	Nasal/10MCL-N
11	52	F	MCL	Arm/11MCL-C	Nasal/11MCL-N
12	25	M	MCL	Trunk/12MCL-C	Oral/12MCL-O
13	52	F	MCL	ND	Oral/13MCL-O
14	54	M	MCL	Upper lip/14MCL-C	Nasal/14MCL-N
15	69	M	MCL	ND	Nasal/15MCL-N
16	65	M	MCL	ND	Oral/16MCL-O
17	63	F	ML	A	Nasal/17ML-N
18	53	M	ML	A	Nasal/18ML-N
19	66	F	ML	A	Nasal/19ML-N
20	71	M	ML	A	Nasal/20ML-N
21	12	M	ML	A	Nasal/21ML-N
22*	49	F	ML	A	Nasal/22ML-N
23*	69	M	ML	A	Nasal/23ML-N
24*	60	M	ML	A	Nasal/24ML-N
25*	77	M	ML	A	Nasal/25ML-N
26*	72	M	ML	A	Nasal/26ML-N
27*	71	F	ML	A	Nasal/27ML-N
28*	39	M	ML	A	Nasal/28ML-N
29*	63	M	ML	A	Nasal/29ML-N
30*	44	F	ML	A	Nasal/30ML-N
31*	31	F	ML	A	Nasal/31ML-N1
					Nasal/31ML-N2
32*	29	F	ML	A	Nasal/32ML-N1
					Nasal/32ML-N2
33*	72	F	ML	A	Oral/33LM-O
34	49	M	DL	Arm/ 34DL-C1	Nasal/34DL-N
				Trunk/34DL-C2	
				Leg/34DL-C3	
35	41	M	DL	Nose/35DL-C	Nasal/35DL-N
					Oral/35DL-O

*, Patients reporting the absence of an initial cutaneous lesion or scar suggestive of leishmaniasis; *M *male, *F *female, *MCL*, mucocutaneous leishmaniasis, *ML *mucosal leishmaniasis, *DL *disseminated leishmaniasis, *ND *not done, *A *absence of cutaneous lesion at the time of clinical evaluation.

### Molecular diagnostics and analysis of genetic variability

From the 35 patients, 52 tissue samples were analyzed, of which 13 originated from cutaneous lesions and 39 from mucosal lesions. Samples from cutaneous and mucosal lesions were only available from 9 of the 16 patients with MCL. From 2 of the patients with ML (patients 31 and 32), fragments of the mucosal nasal lesions were collected at different times over a period of one year. From patient 34 with DL, three samples of cutaneous lesions were analyzed, two of which were collected at the same time as the sample of the mucosal lesion and one that was collected 6 months after treatment.

Internal controls for PCR inhibition were performed by amplifying a 110 bp fragment of the human β-globin gene. The expected 110 bp fragment was obtained in each sample tested (data not shown), confirming the absence of internal inhibitors. The 750 bp diagnostic band was evident in all samples analyzed (data not shown).

**Figure 1:**
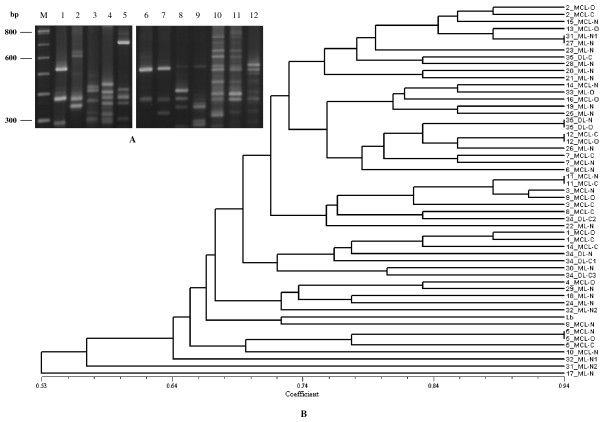
**kDNA signatures of *L. (V.) braziliensis *obtained by LSSP-PCR and phenetic analysis. A: **1.8% High Resolution agarose gel electrophoresis showing representative genetic profiles derived from amplification of the 750 bp fragment from mucosal lesions of different patients. Lanes: M – Molecular marker (100 bp ladder); lanes 1–5: 22ML-N, 26ML-N, 29ML-N, 4MCL-O and 16MCL-O; lanes 6–12: 11MCL-N, 11MCL-C, 3MCL-N, 3MCL-C, 5MCL-N, 5MCL-O and 5MCL-C. **B: **UPGMA dendrogram showing the “Simple Matching” coefficients of similarity based on the genetic profiles obtained by LSSP-PCR. Lb, *L*. (*V*.) *braziliensis *reference strain (MHOM/BR/1975/M2903). MCL: mucocutaneous leishmaniasis; ML: mucosal leishmaniasis; DL: disseminated leishmaniasis; N: nasal; O: oral; C: cutaneous; C1-C3: different samples of cutaneous lesions collected at different moments; N1 and N2: different samples of mucosal nasal lesions collected at different moments.

Different genetic profiles, representing kDNA signatures of the parasite, were obtained by LSSP-PCR analysis of each sample. Figure 1-A shows selected representative results. Phenetic analysis grouped the LSSP-PCR profiles according to their coefficients of similarity (Sm), which diverged from 0.53 to 0.94 (Figure 1-B). The dendrogram illustrates that in two patients with MCL (patients 12 and 11) samples of cutaneous and mucosal lesions were genetically similar to each other, sharing a coefficient of similarity of 0.94. In two other patients, one with MCL (patient 5) and one with DL (patient 35), the nasal and oral mucosal lesions also presented similar genetic profiles (Sm = 0.94), although somewhat differing genetic profiles (Sm = 0.78 and Sm = 0.82, respectively) were apparent in the respective cutaneous lesions. Patients 31 and 27, with the ML clinical condition, also showed similar genetic profiles in the samples of their mucosal nasal lesions. On the other hand, distinct genetic profiles were detected in samples from the cutaneous and mucosal lesions of patients 14, 3, 8 and in the nasal mucosal lesions of patients 31 and 32, which were collected at different times over a period of one year. Interestingly, very divergent profiles were observed in the cutaneous and mucosal lesion samples of patient 34 and also in the mucosal lesion samples of patients 31, 17 and 32.

In order to evaluate interpatient genetic variability, the percentage of shared bands was analyzed. This approach was validated by first demonstrating that, on average, each character (band) was shared by 77% of the patients. Analysis of interpatient coefficients of similarity revealed a similar average of 0.69 (SD = 0.05). On the other hand, the average characters shared at the intrapatient level was 89%, and the average of the average coefficients of similarity was 0.80 (SD = 0.12). Comparison of the average inter and intrapatient coefficients of similarity and their standard deviations were statistically significant (p < 0.001). Table 2 represents the inter and intrapatient values of the coefficient of similarity for selected representative samples.

**Table 2:** **Table showing the values of the intra**- **and interpatient coefficients of similarity of selected representative samples**

	**5MCL**-**C**	**5MCL**-**N**	**5MCL**-**O**	**12MCL**-**C**	**12MCL**-**O**	**34DL**-**N**	**34DL**-**C1**	**34DL**-**C2**	**34DL**-**C3**	**31ML**-**N1**	**31ML**-**N2**	**9MLC**-**O**	**15MCL**-**N**	**23ML**-**N**	**20ML**-**n**	**33ML**-**0**
5MCL-C	1.00															
5MCL-N	0.77	1.00														
5MCL-O	0.77	0.94	1.00													
12MCL-C	0.66	0.72	0.72	1.00												
12MCL-0	0.72	0.72	0.72	0.94	1.00											
34DL-N	0.69	0.58	0.58	0.69	0.69	1.00										
34DL-C1	0.55	0.61	0.66	0.66	0.66	0.75	1.00									
34DL-C2	0.69	0.58	0.63	0.75	0.75	0.77	0.75	1.00								
34DL-C3	0.72	0.72	0.72	0.66	0.66	0.69	0.77	0.75	1.00							
31 M1-N1	0.66	0.72	0.72	0.77	0.77	0.63	0.66	0.63	0.66	1.00						
31ML-N2	0.50	0.55	0.50	0.55	0.61	0.58	0.55	0.52	0.50	0.55	1.00					
9MCL-O	0.72	0.66	0.66	0.77	0.83	0.69	0.55	0.75	0.61	0.72	0.66	1.00				
15MCL-N	0.75	0.63	0.63	0.80	0.80	0.72	0.69	0.72	0.75	0.86	0.58	0.75	1.00			
23ML-N	0.63	0.63	0.63	0.75	0.69	0.66	0.58	0.66	0.58	0.80	0.52	0.69	0.83	1.00		
20ML-N	0.72	0.77	0.77	0.77	0.83	0.63	0.61	0.63	0.61	0.83	0.55	0.83	0.69	0.69	1.00	
33ML-O	0.66	0.61	061	0.77	0.77	0.69	0.67	0.69	0.66	0.72	0.66	0.72	0.80	0.75	0.72	1.00

*MCL* mucocustaneous leishmaniasis, *DL* disseminated leishmaniasis, *ML* mucosal leishmaniasis, *N* nasal lesion, *O* oral lesion, *C* custaneous lesion, *N1* and *N2* nasal lesion fragments collected at 2 different times, *C1*-*C3* cutaneous lesion fragments collected at different times.

### The conceptual model

**Figure 2:**
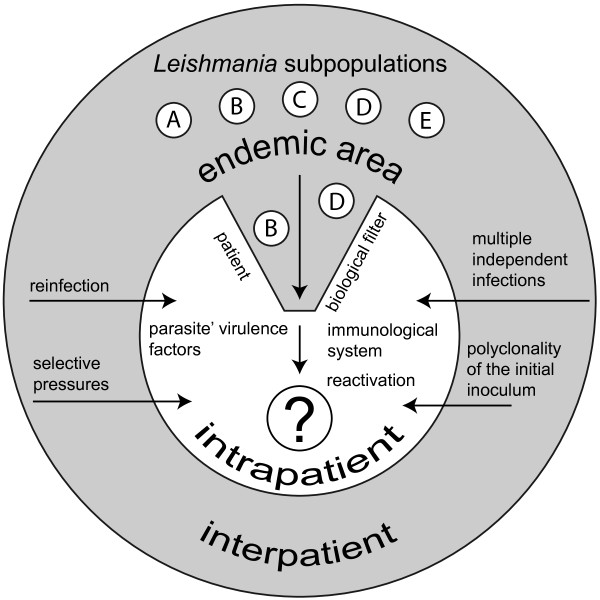
**Conceptual model proposed by Pacheco RS and Oliveira FS aiming to explain the dynamics of the circulating populations of *L. (V.) braziliensis *at both intra and interpatient levels in a specific endemic area. **At interpatient level, heterogeneous subpopulations (A-E) circulate among man, vectors, domestic and sylvatic animals and such individuals would be exposed to distinct selective pressures, to the polyclonality of the initial inoculum and also to multiple independent infections. At intrapatient level, the individual may act as a biological filter and the situation takes into account the participation of both parasite virulence factors and the immunological system of the host.

We have proposed a conceptual model (Figure 2) as a way to explain the dynamics of the circulating populations of *L*. (*V*.) *braziliensis* at both intra and interpatient levels in a specific endemic area.

## Discussion

Our group has recently been applying different molecular tools towards the study of specific diagnostics and the genotypic behaviour of *L*. (*V*.) *braziliensis* subpopulations in patients with mucocutaneous leishmaniasis [20,21]. These studies served as a basis for the present investigation, which aimed to explore the relationship between genetic diversity of *L*. (*V*.) *braziliensis* at inter and intrapatient levels presenting distinct clinical manifestations of ATL. At the same time, this study also aimed to evaluate such genetic diversity following two hypothetical scenarios: (i) the individual, by him/herself, acting as a biological filter whereby highly adapted populations are selected and (ii) eco-epidemiological interactions occurring in the endemic area as reflecting on the dynamics of the circulating populations of *L*. (*V*.) *braziliensis*. According to our conceptual model, the situation at intrapatient level takes into account the participation of both parasite virulence factors and the immunological system of the host and that the reactivation of the disease would reflect in the emergence of different parasite subpopulations. However, at the interpatient level circumstance is that heterogeneous subpopulations circulate among man and vectors as well as domestic and sylvatic animals in simple and complex epidemiological interactions. In such specific cases individuals would be exposed to distinct selective pressures, to the polyclonality of the initial inoculum and also to multiple independent infections. If the biological entity that evolves is the clonal lineage [18], one can imagine different clones being reshuffled and propagated in the natural circulating population, sharing different factors of virulence or competing for selective advantages such as distinct growth rates and differential tropisms [23,24].

Genetic variability of *L*. (*V*.) *braziliensis*, demonstrated here by polymorphisms of kDNA signatures, was identified among the patients and among different lesions of the same patient. Phenetic analysis was adopted to evaluate the degree of heterogeneity of the kDNA minicircles and group them into genetic profiles of different levels of differentiation, from more similar to more divergent. Although this study did not uncover a relationship between parasite genotype and clinical manifestations of ATL, considerably divergent genetic profiles have been detected in nasal mucosal lesions of patients with ML. Schriefer *et al*. [25] noted that a higher frequency of a determined genotype of *L*. (*V*.) *braziliensis* in certain isolates of ML originated from the same endemic area in the state of Bahia.

This idea of multiple independent infections may be an explanation corroborated by data from patient 34, with DL, whose results display significant genetic diversity in the minicircles from distinct cutaneous and mucosal lesions collected both at the same time and also at different moments over a short period of time.

Despite the existence of heterogeneity, genotypic similarities between the samples from cutaneous and mucosal lesions were evident on the intrapatient level. The average of the coefficients of similarities (0.80) was also of major significance, indicating that the samples are genetically related. Therefore, the intrapatient genetic variability, less than that between different patients (77%), must be a result that is also in accordance with the percentage of shared intrapatient characters (89%).

Elegant studies have demonstrated that the host immune system plays an important role in the selection of parasite populations [26-28]. Considering this, it is possible that man may act as a biological filter towards the selection of parasite subpopulations most adapted to develop in the host organism, and in this case, subpopulations can be responsible for either cutaneous or mucosal pathologies as mentioned in our conceptual model.

From the epidemiological point of view, the complex cycle of transmissions in which the individuals are exposed is a very important factor for the possibility of reinfections through the accumulation of multiple independent infections in patients residing in endemic areas [8,29]. The complexity of transmission cycles, involving sylvatic and domestic mammals together with the phlebotamine vectors surely contributes to the large diversity of the natural population of *L*. (*V*.) *braziliensis*[30,31]. An understanding of the genetic diversity of circulating parasite populations in a specific endemic area, as well as an investigation of the clonality of the initial lesions, is fundamentally important and assists us in interpreting the inter and intrapatient variability.

## Conclusion

This study addresses the genetic variability of *L*. (*V*.) *braziliensis*, demonstrated by polymorphisms of kDNA signatures, not only among the patients but also among different lesions of the same patient. Despite the existence of heterogeneity, genotypic similarities between the samples from cutaneous and mucosal lesions were evident at the intrapatient level, the genetic variability being less pronounced than the variability between different patients. A conceptual model was proposed to better understand the complexity at both levels.

## Competing interests

The author declared that they have no competing interest.

## Authors’ contributions

FSO and RSP conceived the study; FSO and RSP designed the study; FSO performed the PCR assays and the genetic characterization of the parasites; CMVR was responsible for othorinolaryngologic evaluation and collection of the biological samples; SJBP and FACC contributed design and statistical analysis; RSP, CMVR and AOS analysed and interpreted the data; FSO drafted the manuscript. RSP critically revised the manuscript for intellectual content. All authors read and approved the final manuscript. FSO and RSP are guarantors of the paper. RSP and AOS are investigators of CNPq, Brazil.
